# Hydrology controls recruitment of two invasive cyprinids: bigheaded carp reproduction in a navigable large river

**DOI:** 10.7717/peerj.3641

**Published:** 2017-09-14

**Authors:** Daniel K. Gibson-Reinemer, Levi E. Solomon, Richard M. Pendleton, John H. Chick, Andrew F. Casper

**Affiliations:** 1 Illinois Natural History Survey, Illinois River Biological Station, Prairie Research Institute, University of Illinois, Havana, IL, United States of America; 2 Department of Natural Resources, Cornell University, Ithaca, NY, United States of America; 3 Great Rivers Field Station, Illinois Natural History Survey, Prairie Research Institute, University of Illinois, Alton, IL, United States of America

**Keywords:** Invasive species, Silver carp, Bighead carp, *Hypophthalmichthys*, Flood, Illinois river, Asian carp

## Abstract

In the Mississippi River Basin of North America, invasive bigheaded carp (silver carp *Hypophthalmichthys molitrix* and bighead carp *H. nobilis*, also referred to as Asian carp) have spread rapidly over the past several decades. In the Illinois River, an important tributary of the Upper Mississippi River, reproduction appears to be sporadic and frequently unsuccessful, yet bigheaded carp densities in this river are among the highest recorded on the continent. Understanding the causative factors behind erratic recruitment in this commercially-harvested invasive species is important for both limiting their spread and managing their harvest. We analyzed weekly catch records from 15 years of a standardized monitoring program to document the emergence of age-0 bigheaded carp in relation to environmental conditions. The appearance of age-0 fish was generally linked to hydrographic attributes, which probably serve as a cue for spawning. However, we found profound differences in the number of age-0 fish among years, which varied by as much as five orders of magnitude in successive years. The strong link between summer flooding and age-0 fish production we observed emphasizes the importance of understanding the hydrologic context in which sustained invasions occur. Despite evidence of sporadic recruitment, bigheaded carp populations in the Illinois River appear to be consistent or increasing because of particularly strong, episodic year classes.

## Introduction

Silver carp *Hypophthalmichthys molitrix* and bighead carp *H. nobilis* (hereafter bigheaded carp) have substantially expanded their range throughout the Mississippi River basin since their unintentional introduction in the 1970s (Kolar et al., 2005; Irons et al., 2009; Cooke, 2016). They are currently documented in 23 of the 31 states comprising the Mississippi River basin, including the Arkansas, Missouri, and Ohio rivers (Baerwaldt & Irons, 2013). Throughout their current range, their population densities vary widely from an observation of a single individual (Klumb, 2007) to an estimated 2,544 individuals per river km or 5.5 metric tons per river km (Sass et al., 2010). Differences in population density may be largely an artifact of distance from original source populations or time since arrival. However, bigheaded carp densities are also influenced by environmental and anthropogenic factors, including water chemistry, harvest, primary and secondary production, climatic and hydrologic parameters, propagule pressure, and hydrologic connectivity (Kolar et al., 2005; Lohmeyer & Garvey, 2009; Murphy & Jackson, 2013; Cooke, 2016).

Although the aforementioned factors influence adult densities, less is known about what conditions lead to the successful spawning and recruitment that are critical to population establishment and persistence. For successful reproduction, bigheaded carp require suitably warm temperatures, a fluctuation in water level, and moderate to swift current to maintain the suspension of eggs until hatching (Krykhtin & Gorbach, 1982; Kolar et al., 2005; Garcia et al., 2013). Because bigheaded carp are an invasive species expanding their range (Cooke, 2016), understanding how hydrology influences reproduction and recruitment is critical in predicting future range expansion (Lohmeyer & Garvey, 2009). For the purposes of our analysis, we do not distinguish between silver carp and bighead carp because there is extensive hybridization between the two species in the Illinois River (Lamer et al., 2015).

**Figure 1:**
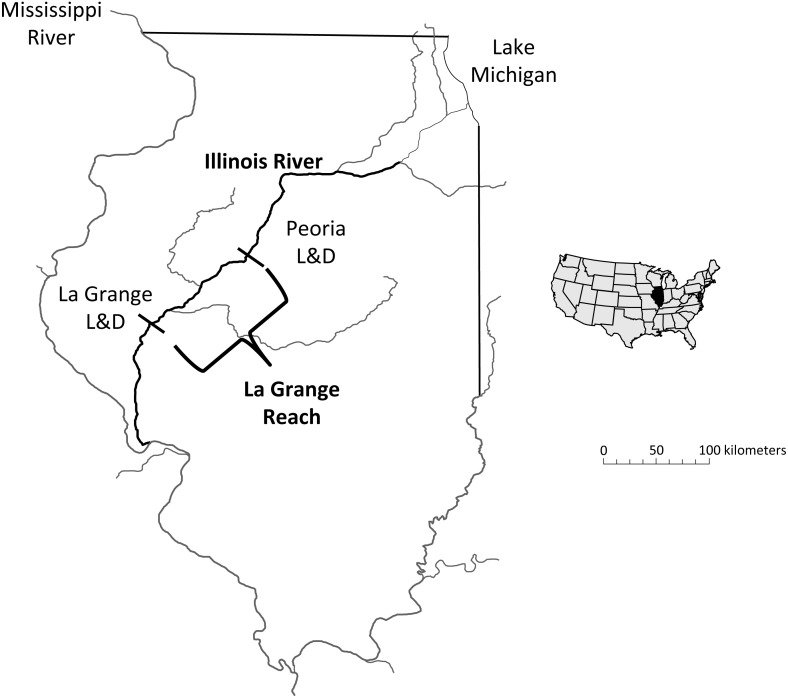
Map of the study area, highlighting the La Grange Reach of the Illinois River. The abbreviation “L & D” indicates a lock and dam.

Within the Mississippi River basin, hydrologic conditions vary spatially due to differences in geomorphology, drainage area, regional precipitation, and the presence of dams or other water control structures (Benke & Cushing, 2005; Theiling & Nestler, 2010). These hydrologic variations can strongly influence the reproductive success and recruitment of bigheaded carp throughout the basin. The Illinois River, a tributary to Mississippi River, is comprised of a series of pooled sections created by eight locks and navigation dams that, along with basin geology and geomorphology, create variable local hydrologic conditions (Fig. 1; Delong, 2005; Lian et al., 2012). Despite major modifications over the past century, the Illinois River is still considered a large, floodplain river with a mosaic of side channels and backwater lakes and sloughs (Delong, 2005). Population densities of bigheaded carp also vary longitudinally throughout the river with densities in the La Grange Reach estimated to be among the highest anywhere (Sass et al., 2010). In addition, the Illinois River has a rich history of long-term monitoring and provides an ideal system to investigate patterns and trends of bigheaded carp reproductive success over time (McClelland et al., 2012; Irons et al., 2011).

In this study, we examined how changes in environmental conditions affected the recruitment of bigheaded carp in a portion of their invaded range that is densely populated. To document how recruitment responded to environmental conditions, we examined data collected during weekly summer monitoring between 2000 and 2014. The period of 2000 to 2014 is notable because age-0 bigheaded carp were first sampled in 2000, so we are able to use the entire known history of reproduction within the reach. We examined both the timing of age-0 bigheaded carp emergence and their abundance across years in relation to factors known to be influential for their spawning. Our objective was to identify when spawning occurred relative to the height of the river, which serves as an indicator of access to floodplain habitat. Specifically, we examined water temperature, stage height, and the rate of change in stage height. Based on our observations of encountering abundant age-0 fish in years with strong floods, we expected the height of the river to be closely associated with abundant bigheaded carp year classes.

## Methods

The Illinois River is a large, navigable, floodplain river commonly broken down into two distinct sections: the upper river upstream of the “big bend” at Hennepin, Illinois (river km 334) and the lower river stretching downstream of the big bend to the confluence with the Mississippi River (McClelland et al., 2012). The lower river flows through a historic channel previously occupied by the Mississippi River characterized by a wide floodplain, silt/sand substrates, and an extremely low gradient (2 cm/km) (Delong, 2005; McClelland et al., 2012). The La Grange Reach of the Illinois River is part of the lower river, stretching from river km 129–254.

The US Army Corps of Engineer’s Upper Mississippi River Restoration Program’s Long Term Resource Monitoring (LTRM) element has consistently sampled the La Grange Reach of the Illinois River since 1993. The LTRM element uses a multi-gear approach (day electrofishing, fyke netting, mini fyke netting, and small and large hoop netting), standardized sampling methods, and a stratified-random sampling design supplemented by selected fixed sites to assess fish population and community characteristics (Ratcliff et al., 2014). Although the design of this monitoring program was derived to provide information on multiple species rather than for a single target species (Ickes, Sauer & Rogala, 2014), the methods appear to sample bigheaded carp effectively and provide valuable data that has been used to document their potential impacts (Irons et al., 2011; Sass et al., 2014; Solomon et al., 2016). The standardization of the sampling design was an important aspect of our analysis. Briefly, fish sampling conducted by the LTRM in the La Grange Reach occurs from June through October during three periods, which are equal in duration and consecutive. Effort for each gear type is also equally distributed among periods (Table 1). Within each sampling period, effort of gear types is allocated randomly with regard to site location and timing (Ratcliff et al., 2014). All fish data used in the analysis are available to download using the US Geological Survey’s website for LTRM sampling (US Geological Survey, 2016). From 2000 to 2014, sampling occurred each week between June 15 and the end of October, with the exception of the first third of this sampling period in 2005 and 2006.

**Table 1:** Distribution of sampling effort across and within years for sampling used in the analysis. The number of day electrofishing samples, mini fyke nets samples, and fyke net samples in each period of each year are listed. P1, P2, and P3 refer to period 1, period 2, and period 3 of LTRM sampling, respectively.

Year	Day electrofishing			Mini fyke nets			Fyke nets		
	P1	P2	P3	P1	P2	P3	P1	P2	P3
2000	41	42	42	29	29	30	14	13	14
2001	42	42	42	30	30	30	14	14	14
2002	42	42	42	30	30	30	14	14	14
2003	40	40	40	28	28	28	12	12	12
2004	40	40	40	28	28	28	12	12	12
2005	0	40	40	0	28	28	0	12	12
2006	0	40	40	0	28	28	0	12	12
2007	40	40	40	28	28	28	12	12	12
2008	39	40	40	28	27	28	12	12	12
2009	40	40	40	28	28	28	12	12	12
2010	40	40	40	28	28	28	12	12	12
2011	40	40	40	28	28	28	12	12	12
2012	40	41	40	28	28	28	12	12	12
2013	40	40	39	28	28	28	12	12	12
2014	40	40	40	28	28	28	12	12	12

To monitor the appearance and growth of age-0 fish while reducing bias, we used several approaches. First, the distribution of sampling effort was equal in all three periods and took place with highly standardized methods. For example, daytime electrofishing occurred with a targeted effort of 15 min, and mini fyke nets were set for 24 h. Second, the rapid growth of bigheaded carp can create difficulties if a single gear type is used to track their growth, as some age-0 bigheaded carp can reach total lengths of 300 mm within their first summer of life (Irons et al., 2011). All gears have some bias in the size of the fish they sample (e.g., Neumann & Allen, 2007). As age-0 fish increase in size from June to October, the size-selective nature of any single gear would inadequately sample the growing fish. Therefore, we combined catch data from all gears in our analysis to provide a large sample size that reduced size-selective gear bias. Water temperature data was measured during each sampling occasion. We used the mean weekly water temperature from all sampling occasions to assess which weeks were thermally suitable for spawning, using the 18 °C threshold (Cooke, 2016). Our analyses included data from fish captured by daytime electrofishing, mini-fyke nets, and fyke nets, which were used throughout the period, as well as seining, which was used only in 2000 and 2001 (all catch data are available in File S1).

To identify dominant year classes, we used the strong pattern of high reproduction, as indicated by the amount of age-0 fish captured in a year, followed by recruitment of such cohorts into the adult population in subsequent years. We analyzed LTRM sampling data summed by week to provide sufficient temporal resolution for illustrating spawning and recruitment trends. To track the emergence of age-0 fish and to examine for multiple spawning events within years, we plotted weekly histograms of bigheaded carp lengths for fish smaller than 200 mm TL. For fish less than 200 mm TL, we combined all gear types. Although some age-0 bigheaded carp exceeded 200 mm TL in 2000, the first year age-0 fish were detected in our samples, subsequent cohorts do not appear to have grown as swiftly (Irons et al., 2011). We examined how closely spawning events tracked hydrologic conditions, specifically the height of the river and how quickly it was rising. To reduce the possibility that unsuitably cool temperatures were limiting spawning, we did not consider data collected in weeks in which the mean water temperature was below 18 °C (water temperature data used in this study are available in File S2).

To estimate weeks in which spawning of observed age-0 bigheaded carp occurred, we estimated the hatch date from estimates of age-0 growth rates, as hatching occurs one day after spawning (Jennings, 1988). We used the estimates for the size at hatching (6 mm) and daily growth rate (2.24 mm/d) derived from a study that used lapilli otoliths of age-0 silver carp on the Mississippi River to derive daily growth estimates (Michael Wolf, Minnesota Department of Natural Resources, pers. comm., 2016). This estimate of daily growth rates of age-0 fish is the only one we are aware of in the region, and conditions for growth of age-0 are relatively similar in the Middle Mississippi River and the Illinois River. We were able to track the growth of age-0 fish throughout the year by examining weekly histograms of the size distributions of small fish captured. In the years we examined, a single cohort of fish usually dominated the composition and could be tracked growing in size across weeks. From these cohorts, we used the fish captured earliest, and therefore closest to their hatch dates (at ∼10 − 30 mm TL) to derive estimated hatch dates. In some years, an additional cohort emerged at a different time, and we were able to distinguish these two groups based on size differences.

We used the hydrologic data from a USGS gaging station in the La Grange Reach to characterize the hydrologic conditions of the portion of the river sampled by LTRM (gauge 05568500 at Kingston Mines, IL; data available in File S3). For each year, we examined the hydrologic conditions present in the week of estimated spawning and the week preceding this. Specifically, we used two main metrics to describe changes in the hydrograph: (1) the maximum stage height; and (2) the maximum three-day mean change in stage height. Weeks in which spawning occurred were identified using back-calculated hatch dates, described above. To examine the influence of maximum stage height and the rate of change in stage height, we created a scatterplot using data from each week in which water temperatures were above 18 °C (data available in File S4). For the purpose of identifying flood stage, we used a designation of 4.27 m (14 feet; National Weather Service, 2016). We used a χ^2^ test to assess whether spawning was more prevalent in weeks in which the river height exceeded flood stage (chisq.test in R; R Core Team, 2016).

## Results

**Figure 2:**
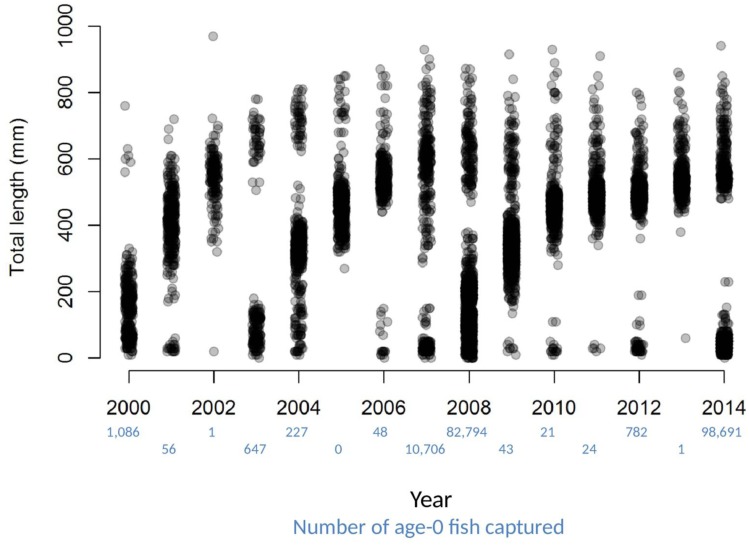
Total lengths of bigheaded carp captured by the LTRM sampling. The *x*-axis has been jittered to avoid overplotting. The number of age-0 fish captured in each year is listed in blue text beneath the year.

Dramatic variations in the number of age-0 fish sampled each year, combined with patterns of size distributions over time, provide striking visual evidence of a fish population dominated by sporadic recruitment (Fig. 2). Age-0 fish were captured using several gears, but mini-fyke nets (74.1% of the total catch) and daytime electrofishing (25.7% of the total catch) captured the vast majority (all catch data for bigheaded carp used in this study are available in File S2). Over the 15 years of catch data, there appear to be four major clusters of reproduction that sustain the population of bigheaded carp: the 2000 year-class, the combination of the 2003 and 2004 year-classes, the combination of the 2007 and 2008 year-classes, and the 2014 year class. Although there were some age-0 fish captured in most years, sampling data suggest the differences in age-0 abundance between strong year-classes and weak year-classes were substantial. For instance, in 2007 and 2008, there were over 800 times as many age-0 fish captured as in 2005–2006 and 2009–2013 combined. In 2013, only a single age-0 fish was captured, while the number individuals captured in 2014 was nearly five orders of magnitude higher and the highest among all years examined.

**Figure 3:**
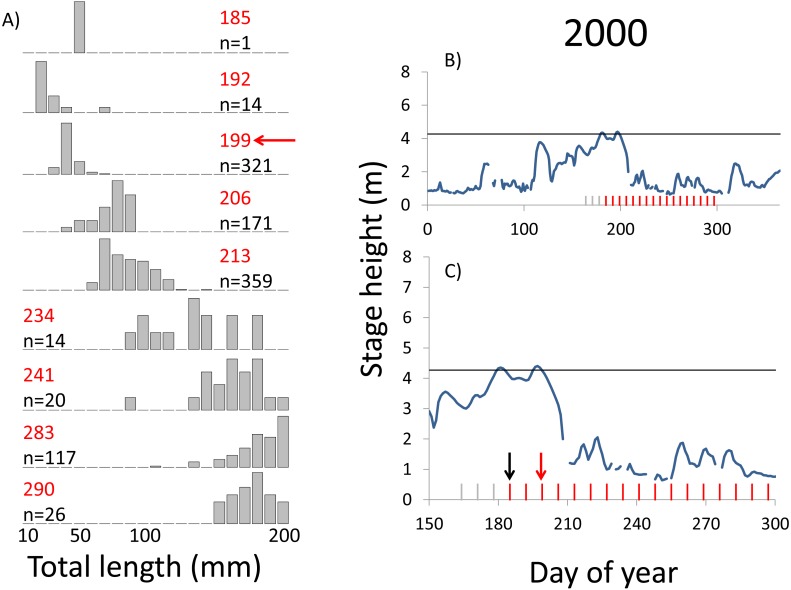
Hydrographs of the Illinois River in 2000 and histograms depicting the size distribution of age-0 bigheaded carp captured in different weeks (A). The ticks on the *x*-axis of the hydrographs represent weeks in which LTRM sampling was conducted. Stage height over the entire year is depicted in the top hydrograph (B), and the bottom hydrograph (C) focuses on the period of fish sampling. Gray ticks indicate there were no age-0 bigheaded carp detected, whereas red ticks indicate age-0 bigheaded carp were detected. Histograms are shown in descending chronological order. Red numbers next to each histogram show the day of the year for the Monday of the week in which sampling began. The numbers in black, beneath the day of year indication, show the number of age-0 bigheaded carp collected. Red arrows indicate weeks with high numbers of age-0 bigheaded carp sampled that were used for back-calculating hatch dates. Black arrows indicate weeks in which spawning was estimated to have occurred. The horizontal black bar depicts the flood stage (4.27 m).

Water temperatures were typically above the threshold for spawning throughout the period in which LTRM samples are collected (mid-June through October). Weekly mean water temperatures fell below the 18 °C threshold after the 40th week of the year (the last week of September), so we did not consider hydrologic data collected after this point. Mean weekly water temperatures when the LTRM sampling began in mid-June were never below the 18 °C threshold. Sampling effort was consistent among years. The mean duration of electrofishing was 15.0 min (SD = 0.19 min). The mean duration of mini-fyke net sets was 23.2 h (SD = 1.59 h).

**Figure 4:**
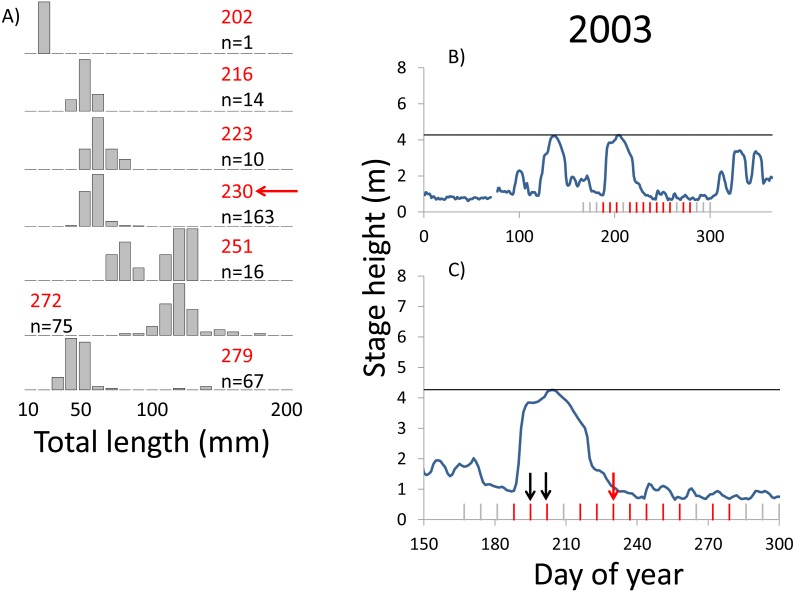
Hydrographs of the Illinois River in 2003 and histograms depicting the size distribution of age-0 bigheaded carp captured in different weeks (A). The ticks on the *x*-axis of the hydrographs represent weeks in which LTRM sampling was conducted. Stage height over the entire year is depicted in the top hydrograph (B), and the bottom hydrograph (C) focuses on the period of fish sampling. Gray ticks indicate there were no age-0 bigheaded carp detected, whereas red ticks indicate age-0 bigheaded carp were detected. Histograms are shown in descending chronological order. Red numbers next to each histogram show the day of the year for the Monday of the week in which sampling began. The numbers in black, beneath the day of year indication, show the number of age-0 bigheaded carp collected. Red arrows indicate weeks with high numbers of age-0 bigheaded carp sampled that were used for back-calculating hatch dates. Black arrows indicate weeks in which spawning was estimated to have occurred. The horizontal black bar depicts the flood stage (4.27 m).

**Figure 5:**
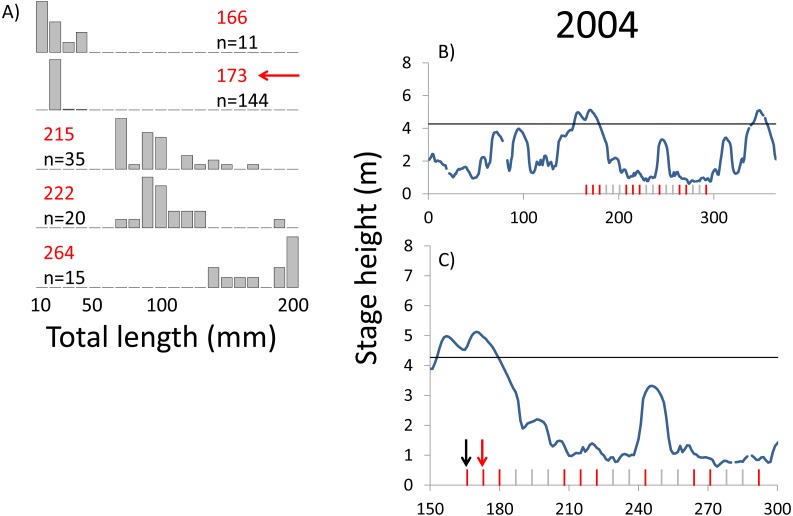
Hydrographs of the Illinois River in 2004 and histograms depicting the size distribution of age-0 bigheaded carp captured in different weeks (A). The ticks on the *x*-axis of the hydrographs represent weeks in which LTRM sampling was conducted. Stage height over the entire year is depicted in the top hydrograph (B), and the bottom hydrograph (C) focuses on the period of fish sampling. Gray ticks indicate there were no age-0 bigheaded carp detected, whereas red ticks indicate age-0 bigheaded carp were detected. Histograms are shown in descending chronological order. Red numbers next to each histogram show the day of the year for the Monday of the week in which sampling began. The numbers in black, beneath the day of year indication, show the number of age-0 bigheaded carp collected. Red arrows indicate weeks with high numbers of age-0 bigheaded carp sampled that were used for back-calculating hatch dates. Black arrows indicate weeks in which spawning was estimated to have occurred. The horizontal black bar depicts the flood stage (4.27 m).

**Figure 6:**
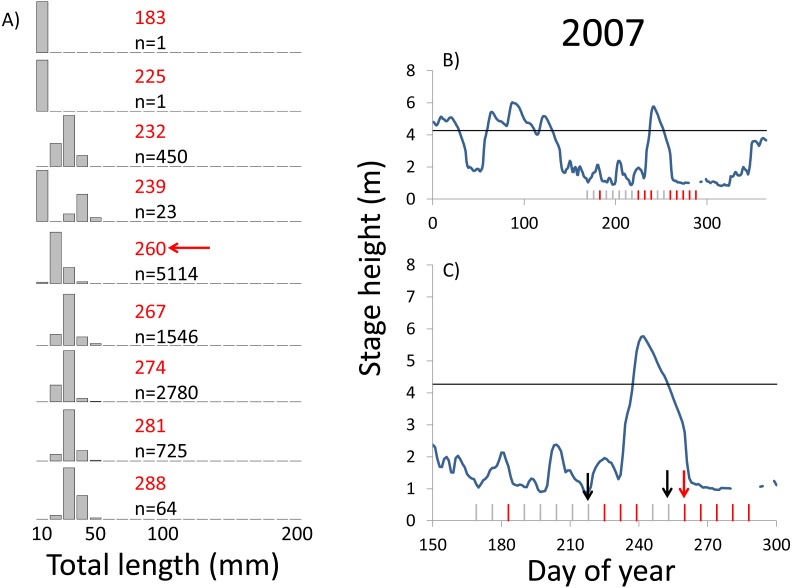
Hydrographs of the Illinois River in 2007 and histograms depicting the size distribution of age-0 bigheaded carp captured in different weeks (A). The ticks on the *x*-axis of the hydrographs represent weeks in which LTRM sampling was conducted. Stage height over the entire year is depicted in the top hydrograph (B), and the bottom hydrograph (C) focuses on the period of fish sampling. Gray ticks indicate there were no age-0 bigheaded carp detected, whereas red ticks indicate age-0 bigheaded carp were detected. Histograms are shown in descending chronological order. Red numbers next to each histogram show the day of the year for the Monday of the week in which sampling began. The numbers in black, beneath the day of year indication, show the number of age-0 bigheaded carp collected. Red arrows indicate weeks with high numbers of age-0 bigheaded carp sampled that were used for back-calculating hatch dates. Black arrows indicate weeks in which spawning was estimated to have occurred. The horizontal black bar depicts the flood stage (4.27 m).

**Figure 7:**
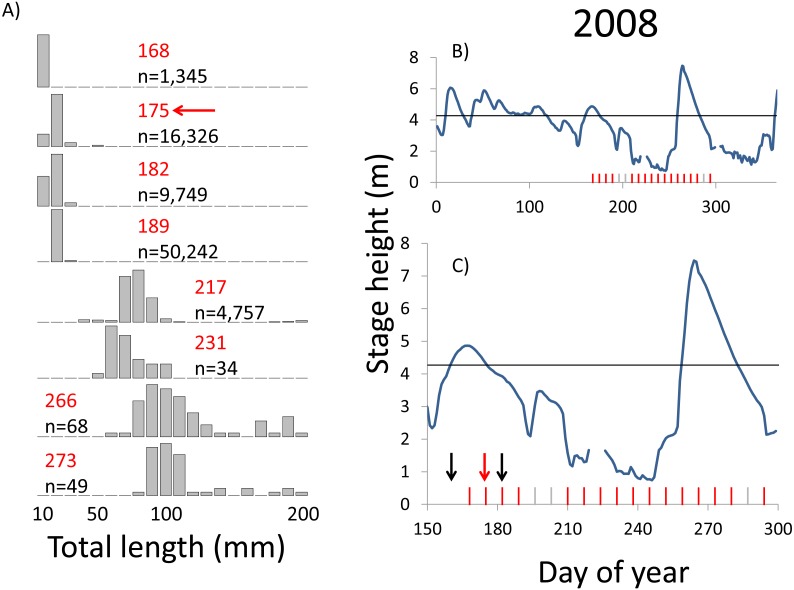
Hydrographs of the Illinois River in 2008 and histograms depicting the size distribution of age-0 bigheaded carp captured in different weeks (A). The ticks on the *x*-axis of the hydrographs represent weeks in which LTRM sampling was conducted. Stage height over the entire year is depicted in the top hydrograph (B), and the bottom hydrograph (C) focuses on the period of fish sampling. Gray ticks indicate there were no age-0 bigheaded carp detected, whereas red ticks indicate age-0 bigheaded carp were detected. Histograms are shown in descending chronological order. Red numbers next to each histogram show the day of the year for the Monday of the week in which sampling began. The numbers in black, beneath the day of year indication, show the number of age-0 bigheaded carp collected. Red arrows indicate weeks with high numbers of age-0 bigheaded carp sampled that were used for back-calculating hatch dates. Black arrows indicate weeks in which spawning was estimated to have occurred. The horizontal black bar depicts the flood stage (4.27 m).

**Figure 8:**
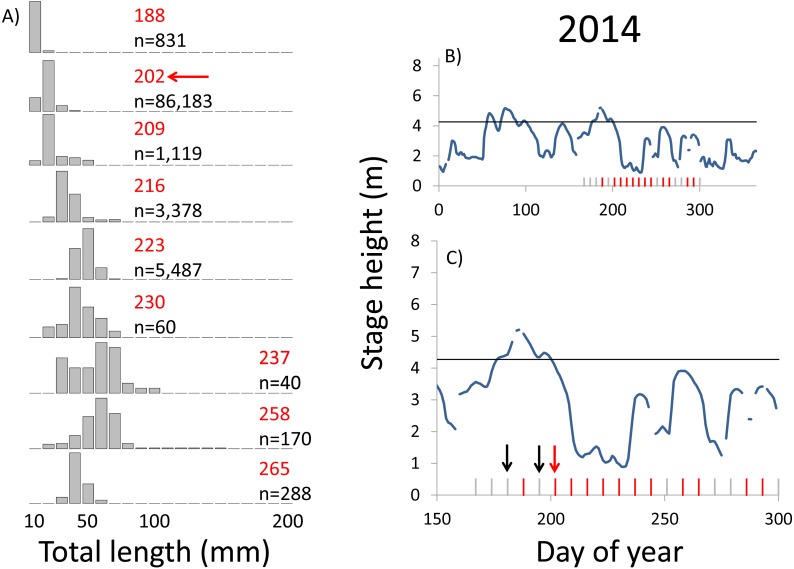
Hydrographs of the Illinois River in 2014 and histograms depicting the size distribution of age-0 bigheaded carp captured in different weeks (A). The ticks on the *x*-axis of the hydrographs represent weeks in which LTRM sampling was conducted. Stage height over the entire year is depicted in the top hydrograph (B), and the bottom hydrograph (C) focuses on the period of fish sampling. Gray ticks indicate there were no age-0 bigheaded carp detected, whereas red ticks indicate age-0 bigheaded carp were detected. Histograms are shown in descending chronological order. Red numbers next to each histogram show the day of the year for the Monday of the week in which sampling began. The numbers in black, beneath the day of year indication, show the number of age-0 bigheaded carp collected. Red arrows indicate weeks with high numbers of age-0 bigheaded carp sampled that were used for back-calculating hatch dates. Black arrows indicate weeks in which spawning was estimated to have occurred. The horizontal black bar depicts the flood stage (4.27 m).

**Figure 9:**
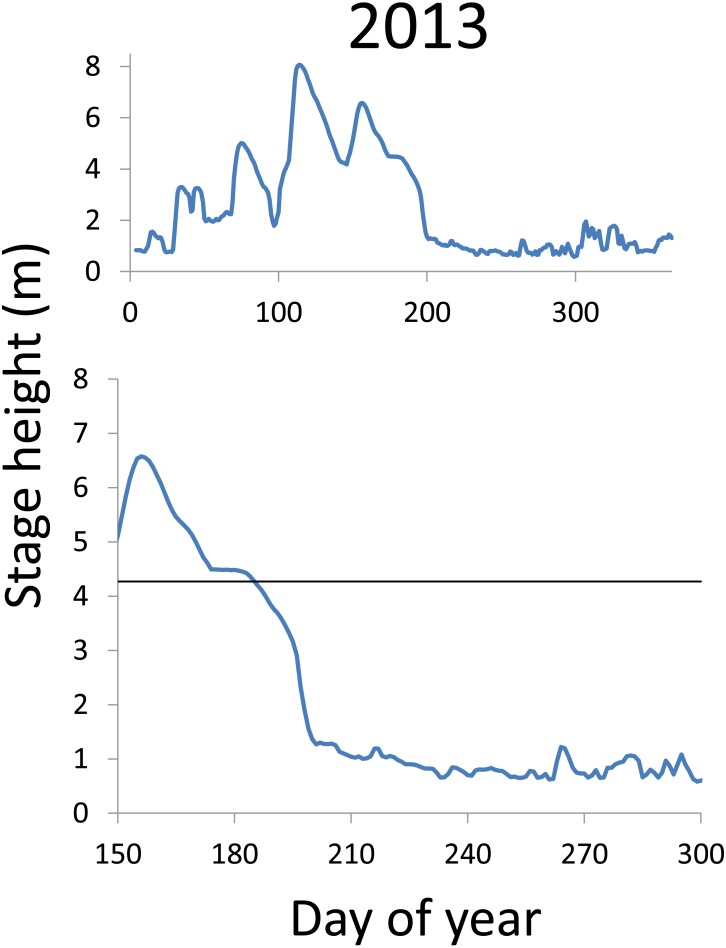
Hydrographs of the Illinois River in 2013. There was only a single age-0 bigheaded carp captured in 2013. The horizontal black bar depicts the flood stage (4.27 m).

**Figure 10:**
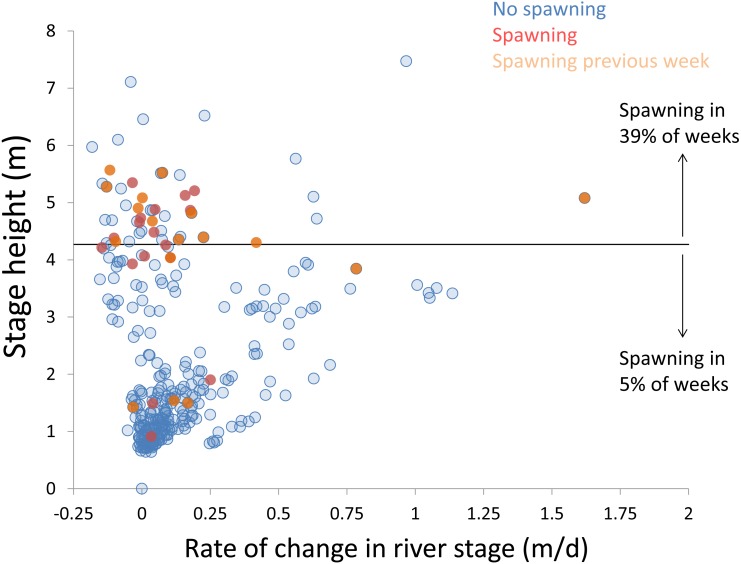
Hydrologic variables influencing bigheaded carp spawning across years. Scatterplot depicting stage height (m) and rate of change in the river stage height (m/d) in weeks without detected spawning (blue points), with detected spawning (red points), and with spawning detected the previous week (orange points). The horizontal black bar depicts the flood stage (4.27 m).

Within each year that produced strong recruitment, the timing of spawning aligns closely with similar hydrologic conditions. In 2000, small fish (∼20 mm TL) appeared shortly after the river rose to near flood stage (Fig. 3). In 2003, a sharp increase in river height to near flood stage was again followed by the appearance of age-0 bigheaded carp (Fig. 4). A flood in 2004 produced a similar result (Fig. 5). In 2007, there was little evidence of strong recruitment early in the year, but a large number of 20 mm fish were captured after a late-summer flood (Fig. 6). In 2008, the large numbers of 10 and 20 mm fish that were captured also followed a flood (Fig. 7). In 2014, the largest number of age-0 fish captured in a single week appeared shortly after another flood (Fig. 8). Not all years with high floods had evidence of recruitment. In 2013, a large flood occurred, yet only a single age-0 bigheaded carp was detected (Fig. 9). The hydrologic conditions in which these strong year classes emerged tended to be when the river was near or above flood stage. Specifically, weeks in which spawning occurred generally displayed high water levels, although the rate at which water levels rose did not appear to be influential (Fig. 10). Evidence of spawning was less frequent below flood stage (4.27 m) and rarely detected when the river height was below 4 m, which constituted the majority of the weeks sampled. When the gage height exceeded 4.27 m, there was evidence of spawning in 39% of weeks (21 out of 54), whereas there was evidence of spawning in only 5% of weeks (13 out of 276) in which water levels were below 4.27 m (χ^2^ = 53.5, *p* < 3∗10^−13^; Table 2).

## Discussion

We observed dramatic differences in the number of age-0 bigheaded carp captured across 15 years of a standardized monitoring program. The methods by which the fish were sampled were highly consistent across each year, summarized in Table 1 and in greater detail in Ratcliff et al. (2014). Because the effort was so consistent, and because we were able to combine catch data across gears to minimize gear bias, we had a powerful sampling design to detect the timing of spawning events that led to recruitment. Six of the 15 years we examined accounted for 99.5% of all age-0 bigheaded carp captured (2000, 2003–2004, 2007–2008, and 2014). Within each of these six years, the size distributions of weekly catch data showed that each year was dominated by a discrete spawning event, as indicated by the shift in size distributions in Figs. 3–8. Back-calculating the hatching date from small fish allowed us to identify the weeks in which spawning that produced age-0 fish occurred and the extent of flooding in each of those weeks. The strong link between flooding and spawning events in the years that produced more than 99% of all age-0 bigheaded carp encountered demonstrates the importance of flooding for bigheaded carp recruitment in the La Grange Reach of the Illinois River.

It is noteworthy that bigheaded carp in the La Grange Reach, the densities of which are among the highest recorded anywhere (Sass et al., 2010), do not have high recruitment in most years. There have been large numbers of reproductively mature fish in the La Grange Reach since 2000, and females can produce 10^6^ − 10^7^ eggs (Cooke, 2016). Despite this abundant potential for reproduction, recruitment is only intermittently successful and apparently dominated by a minority of year classes (2000, 2003–2004, 2007–2008, and 2014). Why is there such extreme fluctuation in reproductive success across years? The magnitude of interannual reproductive variation, and its alignment with flood conditions, suggests the most important mechanisms are not biotic.

Hydrographic records of each year with high recruitment share the same feature of exceeding flood stage (4.27 m). In all but one year (2005, when no age-0 bigheaded carp were detected), the presence of at least some larval and juvenile bigheaded carp provides evidence that they reproduced (Fig. 2). However, it appears the age-0 fish captured in other years make only minor contributions to the population. Field observations of large schools of small (<20 mm) bigheaded carp in 2011, when there was little evidence of heavy recruitment to larger sizes, suggests bigheaded carp may spawn in substantial numbers without producing many recruits (L Solomon, pers. obs., 2011). Thus, although it may be possible for bigheaded carp to reproduce in the La Grange Reach every year, the more important question is to examine why some years are so much more productive than others. Particularly for an invasive species with a rapidly growing population, the question is not whether reproduction occurs in an absolute sense but what creates the high levels of reproduction and recruitment that lead to exponential population growth. Similarly, our analysis focused not on conditions that led to spawning in an absolute sense but the spawning events that created strong cohorts of fish.

Years with differences in the timing of flooding provide additional insight into the factors most responsible for spawning and recruitment of bigheaded carp in the Illinois River. In 2007, more age-0 fish were captured than in all previous years combined. This large cohort appeared following a large flood that occurred unusually late, in September, when there had been little recruitment earlier in the summer. The close relationship between hydrology and the emergence of age-0 fish across years with different flood timing, combined with the virtual absence of recruitment in most years, suggests flooding is a more important cue for successful spawning than photoperiod or temperature alone. Temperatures in the La Grange Reach appear to be suitable for reproduction throughout most of the summer across years (i.e., 18–27 °C; Cooke, 2016). Recruitment does not appear to occur from spawning in most of the sampling weeks, suggesting that although temperatures are sufficient, some other factor or factors or insufficient to induce successful spawning. However, recruitment to the adult population may be more sensitive to temperatures, as the fish spawned late in 2007 would have had a much shorter period of optimal growth conditions before entering their first winter. We also noted that, although 27 °C has been listed as an upper limit for bigheaded carp spawning, we estimated that spawning occurred in some weeks in which temperatures were as high as 30−31 °C (File S3).

There is a strong association between flooding and the appearance of young bigheaded carp, a relationship that is significant both biologically and statistically. When the river reaches flood stage, successful spawning (as measured by the appearance of age-0 fish) is about eight times more likely to occur than when the river is below flood stage (Fig. 10). Statistically, the large χ^2^ value indicates one of the cell values differs from expectations; in this case, it is the large number of weeks in which spawning and flooding co-occurred (Table 2). Although years with strong recruitment share similar hydrologic conditions, the absence of strong reproduction in some years is more complex. In some instances, the hydrology seems clearly unfavorable: in 2005, the largest flooding occurred in the winter, and the river was unusually low for the entire summer. However, in other instances, there is no detected recruitment despite favorable hydrology and temperature. In 2013, a large flood (>5 m) occurred in early June, accompanied by suitable water temperatures, yet only a single age-0 bigheaded carp was captured. We cannot fully explain the absence of recruitment in some years that appear to be favorable. In 2013, the population of bigheaded carp would have been largely composed of individuals from the 2007–2008 cohort that would have been reproductively mature; in fact, it was this same cohort that produced the largest group of age-0 fish a year later in 2014. Although migration to and from the study area is possible, the persistently large numbers of adults captured each year suggests migration did not play a substantive role in the patterns we observed.

Because our conclusions are based on changes in the number of age-0 fish captured in each year, an important assumption is that changes in sampling efficiency do not confound the relationships between hydrology and abundance. Sampling effort, which was highly consistent across years, cannot account for any meaningful differences among years, but factors that affected sampling efficiency could have had an effect on interannual differences in catches. Variation in environmental conditions undoubtedly creates variations in the detectability and capture efficiency of fish, particularly age-0 fish. However, we noted abundant age-0 fish in years with the strong floods, the very conditions that generally decrease the detectability of fishes. If detectability was low because of flooding, but fish were nevertheless abundant, this should not seriously affect the conclusions. Similarly, the years in which there were few or no age-0 bigheaded carp detected were generally years in which water levels were generally low, and these are the conditions under which detectability might be expected to be higher. Moreover, the changes we observed between years were staggering—in some cases, as much as five orders of magnitude in successive years. Ultimately, our analysis is more categorical in that we are classifying years as successful or unsuccessful, a distinction that does not require high precision. No sampling was conducted during the first third of the sampling season in 2005 and 2006. If there were spawning events during that time that produced large numbers of age-0 fish, we would not be able to detect them; however, if these fish survived beyond the first part of the summer, we should have detected fish later in the year. The absence of large numbers of age-0 fish the later sampling seasons of 2005 and 2006 suggests any bigheaded carp that were spawned earlier in the year did not survive.

**Table 2:** Spawning in weeks with and without flooding during LTRM sampling.

	Spawning	No spawning
Flooding	21	33
No flooding	13	263

Identifying the weeks in which age-0 fish were spawned is also an important aspect of our analysis. The design of the LTRM program, which samples weekly from mid-June through October each year, provides particularly powerful temporal resolution for identifying when spawning of large cohorts occurred. Our estimates of hatch date were also typically based on very small bigheaded carp. In years with the largest cohorts (2004, 2007, 2008, and 2014), we used fish that were 20 mm TL to assess hatch dates. Bigheaded carp in this area can attain lengths of more than 200 mm within a few months (Irons et al., 2011), so the ability to capture these fish at small sizes increased our confidence that they were recently hatched. Additionally, we had the rare opportunity to back-calculate hatch dates using an equation for daily growth rates developed for silver carp, the species most commonly encountered in our analysis, in a nearby area. Although there may be minor variations in growth rates between the Illinois River and the Mississippi River, the fact that silver carp emerge at 6 mm TL and are regularly captured at 20 mm TL reduces the possibility that these differences would substantively affect our results. In a broader sense, the alignment of large clusters of 20 mm silver carp with floods (Figs. 3–8) is a strong measure of the effect of flooding. Even if daily growth rates are substantially different between the Middle Mississippi River and the Illinois River, the difference in our calculations would be only a matter of days for fish that are 20 mm, and this would still place their hatch date during flood conditions, or the rapid rise in river stage that precedes flooding, as flood conditions tend to persist for weeks in this system.

Floods may be particularly important for recruitment of bigheaded carp in rivers with substantial floodplains. The Illinois River has a particularly broad floodplain carved from the ancestral Mississippi River (Delong, 2005). This expansive floodplain, combined with lock and dam structures to maintain a sufficient water depth for navigation (Sparks, Nelson & Yin, 1998), may create a situation in which water flows are only sufficient during floods. The Wabash River in the neighboring state of Indiana serves as a useful contrast because it does not have a floodplain as extensive as the Illinois River. Bigheaded carp in the Wabash River spawn over a prolonged period, showing no apparent correlation with hydrologic conditions (Coulter et al., 2013). Thus, it is possible that rivers with relatively swift flows may provide greater opportunities for bigheaded carp reproduction each year, whereas rivers with larger floodplains and slower flows may be suitable for recruitment in years with large floods. However, we note that Coulter et al. (2013) sampled eggs and larvae, while we sampled small fish, so it is possible prolonged spawning that did not produce appreciable numbers of 20–30 mm fish would not have been detected in our sampling.

There is some evidence that recruitment failures occur because bigheaded carp do not spawn in large numbers in most years, or that spawning occurs but does not produce fish that survive. The LTRM sampling does not include eggs, thereby limiting our ability to assess whether recruitment failures were caused by a lack of spawning, low survival of fertilized eggs, or low survival of larvae. Without knowing whether spawning occurred but was unsuccessful or if spawning did not occur, it is difficult to assess the reasons recruitment failed in most years. However, sampling juvenile fishes has the advantage of being a more direct measure of recruitment. We speculate that female bigheaded carps may resorb their eggs in years without favorable hydrologic conditions, which was documented in a separate reach of the Illinois River in the summer of 2005 (DeGrandchamp, Garvey & Csoboth, 2007). Whatever the mechanism, it is noteworthy that consistently high recruitment is not necessary for bigheaded carp to achieve high population growth rates.

The relationship between hydrology and recruitment of pelagic-spawning cyprinids is mediated by the geomorphic and landscape context in which floods occur. For example, spawning by flathead chub (*Platygobio gracilis*) during large floods in Colorado did not lead to increased recruitment because larval survival was low during floods (Haworth & Bestgen, 2016). In more arid regions, such as Colorado, flooding that is shorter in duration may not provide the advantages offered by longer inundation of floodplains (e.g., King, Humphries & Lake, 2003). In contrast to floods lasting just a few days, floods associated with high recruitment of bigheaded carp on the Illinois River lasted substantially longer and receded more gradually, thereby offering greater access to floodplain resources without the same risk of stranding fish. Although the inundation of the Illinois River’s floodplain is a natural process, there is some evidence that anthropogenic modification of the floodplain (e.g., draining and leveeing) have shifted the timing of flooding. Relative to the 19th century, late 20th century flood patterns in the Illinois River tended to occur later in the year and to produce more flashy hydrographs (Sparks, Nelson & Yin, 1998). We speculate this shift to flooding in warmer months of the year, accompanied by a shorter and more intense floodplain inundation, may have helped bigheaded carp attain exceptional growth rates during their first year (Irons et al., 2011).

The La Grange Reach of the Illinois River is highly suitable for bigheaded carp spawning and recruitment, yet they appear to be sustained primarily through year classes that emerge in a minority of years. The ability of bigheaded carp to achieve tremendous population densities through episodic recruitment suggests they may be capable of invading rivers even when hydrologic conditions appear unsuitable for them in most years. Similar age distributions have been noted in striped bass (*Morone saxatalis*), which also have pelagic egg spawning in large rivers that appears to be related to abiotic factors (Secor, 2000). Climate change will push warmer temperatures upstream rapidly in shallow-gradient rivers (Isaak & Rieman, 2013), which may facilitate the expansion of bigheaded carp. Additionally, changes in streamflow as a consequence of climate change can benefit invasive species (Rahel & Olden, 2008). Over the coming decades, increases in the magnitude or frequency of flooding (e.g., Milly et al., 2002) could allow bigheaded carp to become established in areas that currently lack the conditions they require. Successful invasive species are often adept at adjusting to exploit their local conditions, as bigheaded carp appear to do (Coulter et al., 2013). The plasticity of bigheaded carp reproduction, combined with the risk of increasing flood frequency with climate change (Milly et al., 2002), may create conditions that allow them to expand considerably to the north and west of their current distribution in the Mississippi-Missouri River system. Our observations highlight the need to study how invasive species interact with their environment as well as the value of long-term monitoring to understand the ecology of invasions.

##  Supplemental Information

10.7717/peerj.3641/supp-1 File S1 Catch data used in the analysis Date, species, total length, and catch of bigheaded carps used in the analysis.

10.7717/peerj.3641/supp-2 File S2 Stage height of the Illinois River Raw data on the stage height of the Illinois River used in the analysis.

10.7717/peerj.3641/supp-3 File S3 Temperature data used in the study All temperature data used in the study are presented in this file, which includes measurements taken between 2000 and 2014 in the La Grange Reach of the Illinois River.

10.7717/peerj.3641/supp-4 File S4 Data used to create Fig. 10 This file contains information on the river stage height, the rate of rise in the river stage height, and whether or not spawning was detected.

## Additional Information and Declarations

### Data Availability

The following information was supplied regarding data availability: The data used in this analysis came from the Long-Term Resource Monitoring element of the Upper Mississippi River Restoration Program. This multi-agency program has been sampling the study area with a standardized protocol since 1993. We used their data, which are publicly available, for our analysis. Dryad DOI: 10.5061/dryad.fn4m3.
